# Insights into glycosidic bond specificity of an engineered selective α-L-rhamnosidase N12-Rha via activity assays and molecular modelling

**DOI:** 10.1186/s13568-022-01489-5

**Published:** 2022-11-12

**Authors:** Bo Yu, Shiyu Luo, Yuhan Ding, Zijie Gong, Ting Nie

**Affiliations:** 1 Jiangxi-OAI Joint Research Institute, Nanchang University, Nanchang, 330047 Jiangxi China; 2 College of Chemistry, Nanchang University, Nanchang, 330031 Jiangxi China; 3 Medical College of Dalian University, Dalian, 116622 Liaoning China; 4 State Key Laboratory of Microbial Metabolism, Joint International Research Laboratory of Metabolic & Developmental Sciences, School of Life Sciences and Biotechnology, Shanghai Jiao Tong University, Shanghai, 200240 China

**Keywords:** α-L-rhamnosidase, Catalytic activity, Charge difference

## Abstract

αL-rhamnosidase (EC 3.2.1.40) has been widely used in food processing and pharmaceutical preparation. The recombinant α-L-rhamnosidase N12-Rha from *Aspergillus niger* JMU-TS528 had significantly higher catalytic activity on α-1,6 glycosidic bond than α-1,2 glycosidic bond, and had no activity on α-1,3 glycosidic bond. The activities of hydrolyzed hesperidin and naringin were 7240 U/mL and 945 U/mL, respectively, which are 10.63 times that of native α-L-rhamnosidase. The activity could maintain more than 80% at pH 3–6 and 40–60℃. Quantum chemistry calculations showed that charge difference of the C-O atoms of the α-1,2, α-1,3 and α-1,6 bonds indicated that α-1,6 bond is most easily broken and α-1,3 bond is the most stable. Molecular dynamics simulations revealed that the key residue Trp359 that may affect substrate specificity and the main catalytic sites of N12-Rha are located in the (α/α)_6_-barrel domain.

**Supplementary Information:**

The online version contains supplementary material available at 10.1186/s13568-022-01489-5.

## Introduction

α-L-Rhamnosidase is a hydrolase that specifically releases terminal L-rhamnose groups from various natural glycoside compounds, and it is widely present in bacteria and fungi. It has broad substrate specificity and can hydrolyze α-1,2, α-1,3, α-1,4, α-1,6 and α1-linked glycosidic bonds. α-L-rhamnosidase has been widely used in food processing and pharmaceutical preparation (Wu et al. 2018; Yadav et al. 2010). Li et al. used α-L-rhamnosidase to effectively remove the naringin in citrus juice to remove the bitter taste without changing the attractive aroma of citrus juice (Li et al. 2019). It was also used to hydrolyze the chloropolysporin B containing the terminal L-rhamnose to obtain the antibiotic chloropolysporin C with important clinical value (Yadav et al. 2010). In recent years, α-L-rhamnosidase has also been often used to prepare the prodrug Prunin, which has potential anti-inflammatory and antiviral properties (Yadav et al. 2010).

*Aspergillus niger* JMU-TS528 is a fungus that produces α-L-rhamnosidase with a broad-spectrum of substrate specificity. However, obtaining α-L-rhamnosidase from *Aspergillus niger* JMU-TS528 requires the induction of glycoside-containing rhamnose or terminal rhamnose, and the production process is also regulated by the fungal carbon metabolism inhibition system (Li et al. 2016). In addition, there are many metabolites of *Aspergillus niger*, so multiple purification steps are required, which greatly reduces the recovery rate, thereby, increasing the difficulty of industrial production of natural α-L-rhamnosidase (Li et al. 2020). Obviously, the production of recombinant α-L-rhamnosidase through gene cloning and heterologous expression is a more efficient and economical method. At present, many α-L-rhamnosidase derived from *Aspergillus* have been produced through heterologous expression and have shown excellent property (Yadav et al. 2010). Manzanares et al. expressed the *Aspergillus aculeatus* rhaA gene encoding α-L-rhamnosidase in an industrial wine yeast strain and applied it to grape winemaking. The wine showed a significant increase in the content of aromatic compound linalool (Manzanares et al. 2003). Gerstorferová et al. expressed the α-L-rhamnosidase gene from *Aspergillus terreus* in a Pichia strain. Compared with the natural system, the production time was shorter and the enzyme production increased fourfold (Gerstorferová et al. 2012).

The α-L-rhamnosidase from *Aspergillus niger* JMU-TS528 has been classified in glycoside hydrolase 78 family (GH78) in the carbohydrate-active enzymes (CAZy) database (www.cazy.org). The number of α-L-rhamnosidase GenBank sequences in the GH78 family exceeds 30,000. However, only six crystal structures of α-L-rhamnosidase (BtRha, PDB: 3CIH (Wu et al. 2018), AT-Rha, PDB: 6GSZ (Pachl et al. 2018), RhaB, PDB: 2okx (Cui et al. 2007), KoRha, PDB: 4XHC (O’Neillet al. 2015), SaRha78A, PDB: 3W5M (Fujimoto et al. 2013) and DtRha, PDB: 6I60 (Guillotin et al. 2019)) derived from bacteria were reported. All six α-L-rhamnosidase crystal structures contain a characteristic (α/α)_6_-barrel catalytic domain and several β-sheet domains. Despite some structural information and mutation work has been studied, the key residues that determine the catalytic properties of α-L-rhamnosidase and the molecular mechanism of substrate specificity have not been revealed in the GH78 family (Nghi do et al. 2012; O’Neill et al. 2015).

At the same time, with the development of computational biology theory and the improvement of computer hardware performance, quantum chemistry calculation methods can be used to reveal molecular structure-activity relationships by accurately optimizing and calculating the structural information of biomolecules about bonds and electrons (Dai et al. 2018). In addition, molecular docking and molecular dynamics (MD) simulation methods can be applied to study the interaction between large molecular proteins and small molecular ligands (Cob-Calan et al. 2019). These methods can be used to study the recognition and binding of α-L-rhamnosidase to the substrate, thereby revealing that the key residues for catalysis and the mechanism of substrate specificity. Li et al. examined the conformational flexibility of the catalytic domains by MD simulations, and the results showed that the conformation of the α-L-rhamnosidase mutants, R404S and N578D, were more flexible, which may affect the affinity of α-L-rhamnosidase to the substrate (Li et al. 2020).

Here, we optimized and synthesized the α-L-rhamnosidase gene sequence of *Aspergillus niger* JMU-TS528 by targeting the *P. pastoris* GS115 expression system. The recombinant pPIC9K vector was constructed and transferred into *P. pastoris* GS115 for expression. A recombinant engineered strain N12 with high enzyme activity was screened out, and the enzymatic properties of recombinant α-L-rhamnosidase N12-Rha were further studied. Then, quantum chemistry calculations have been applied to reveal the structure-activity relationship of the substrates from molecular modelling. All-atom MD simulation was used to reveal the interactions between N12-Rha and the substrates.

## Materials and methods

### Strains, plasmids and chemicals

The vector pPIC9K and *Pichia pastoris* GS115 from Sangon (Shanghai, China) were used for protein expression. The culture mediums were purchased from Baoling (Shanghai, China). Rutin, naringin, hesperidin, neohesperidin and myricetin were purchased from Aladdin (Shanghai, China). Methanol and acetic acid were purchased from Macklin (Shanghai, China). Acetonitrile, ethylene diamine tetraacetic acid (EDTA), dithiothreitol (DTT), β-Mercaptoethanol (β-ME),sodium dodecyl sulfate (SDS), dimethylsulfoxide (DMSO), and ethyl acetate were purchased from Xilong (Shantou, China).

### Optimization and synthesis of the Coding Gene

The α-L-rhamnosidase gene sequence of *Aspergillus niger* JMU-TS528 was obtained from GenBank: AGN92963.1 and was optimized by GenScript codon optimization software for expression in *P. pastoris* GS115 (Mauro 2018). The restriction sites for *Eco*R I and *Not* I were located at 5’ and 3’ sites of the coding sequence respectively. The designed gene sequence of α-L-rhamnosidase (Supplementary material, GenBank: KC750908.1) was synthesized by Genscript (Nanjing, China).

### Subcloning, culture and protein expression

The α-L-rhamnosidase encoding sequences was subcloned into pPIC9K vector. The recombinant plasmid was transformed into *P. pastoris* GS115, then the engineered strains were cultured in 25 mL of sterilized buffered glycerol-complex medium (BMGY) at 28 ℃, 220 rpm for 18 h. The cells were collected by centrifugation at 8000 rpm for 10 min. The cells were resuspended and cultured at 28 ℃ and 220 rpm, and methanol (100%) was added to the medium at a concentration of 0.5% (v/v) every 24 h (Wang et al. 2019). Samples were taken at 24 h, 48 h, 72 h, 96 h, 120 h, 144 h, 168 h, and 192 h and stored at − 80 ℃.

### Screening for Engineered strains with high α-L-rhamnosidase activity

A unit of enzyme activity is defined as the amount of enzyme required to hydrolyze 1 µg rutin per minute at pH 4.8 and 50 ℃. Rutin solutions with different concentrations: 250 µg/mL, 500 µg/mL 1000 µg/mL, 2000 µg/mL, 3000 µg/mL, 4000 µg/mL and 5000 µg/mL were prepared. The activity of α-L-rhamnosidase was evaluated by mixing 100 µL enzyme solution and 500 µL acetic acid and sodium acetate buffer (pH 4.8) at 50 ℃ for 10 min, then 900 µL rutin (2 mg/mL) was added. The reaction solution was incubated at 50 °C for 10 min, then keeping it in boiling water for ten minutes to stop the reaction and the samples were analyzed by high performance liquid chromatography (HPLC) (Lim et al. 2015).

### Purification of the recombinant α-L-rhamnosidase and SDS-PAGE analysis

After 192 h of cell culture, the supernatant was collected at 4 ℃, 8000 rpm for 10 min. The crude enzyme solution was fractionated with 75% (NH_4_)_2_SO_4_ at 4 °C for 24 h. Then protein precipitation was dissolved with 0.01 mM PBS (pH 7.0). The protein solution was then added to Ni column with His-tag Affinity Gel. The unbound fractions were removed by washing with 10 column volumes of the binding buffer (pH 7.0) and the bound proteins were eluted with 20 mM elution buffer (pH 7.0). The purified recombinant proteins were treated with N-glycosidase F at 4 °C for 16 h to remove the asparagine-bound N-glycans. Furthermore, the elution buffer was analyzed using 10% SDS-PAGE (Li et al. 2019).

### Substrate specificity and affinity of recombinant α-L-rhamnosidase

The five substrates (naringin, rutin, hesperidin, neohesperidin and myricetrin) were prepared as 1 mg/mL solutions and scanned at full wavelength, respectively. The peak time of the prepared standard substrate was measured by HPLC according to its optimal absorption wavelength (Kim et al. 2016). The reaction solutions were composed of 100 mL α-L-rhamnosidase, 900µL substrate and 500 µL acetic acid and sodium acetate buffer (pH 4.8), and incubated at 50 ℃ for 1 h. After heating at 100 ℃ for 10 min, the reaction solutions were analyzed by HPLC.

### Determination of the pH and the temperature stability

In order to determine the optimal pH and temperature of the recombinant α-L-rhamnosidase, 1 mg/mL naringin was set as the substrate standard and the reaction solutions were composed of 100µL α-L-rhamnosidase, 1000µL substrate and 900 µL acetic acid and sodium acetate buffer (pH 4.8). The pH of the reaction system was set to 3.0, 4.0, 4.2, 4.4, 4.6, 4.8, 5.0, 6.0, 7.0, 8.0, and incubated at 50 °C for 10 min, respectively. In addition, the reaction system with pH 4.8 was incubated at different temperatures (20, 30, 40, 50, 60, 70, 80 °C) for 10 min.

In order to determine the pH and the temperature stability of the recombinant α-L-rhamnosidase, the mixture of enzyme solution and buffer solution was set at a series of pH value of 3.0, 4.0, 4.2, 4.4, 4.6, 4.8, 5.0, 6.0, 7.0, 8.0, which was stored at 4 ℃ for 24 h. Meanwhile, the thermal stabilities of recombinant α-L-rhamnosidase were investigated by keeping the enzyme at 20, 30, 40, 50, 60, 70, and 80 °C for 2 h, respectively (Koseki et al. 2008). Then, the α-L-rhamnosidase activity was determined according to the above HPLC method. The highest enzyme activity was defined as 100%.

### Effects of metal ions, effectors and organic solvents on recombinant α-L-rhamnosidase

In order to explore the influence of exogenous components on the activity of the recombinant α-L-rhamnosidase, we mixed the different metal ions, effectors, and organic solvents (Fig. 3) with 500 ug/mL naringin. The concentration of metal ions was set at 1 mM, 10 mM, 50 mM, 100 mM and 150 mM. The concentration of effectors was set at 1 mM, 10 mM, 50 mM and 100 mM. The proportion of methanol and DMSO in the water phase were 2.5%, 5%, 10%, 15%, 25%, 30%, respectively (Li et al. 2016). The above solutions were added to the α-L-rhamnosidase buffer (pH 4.8) and incubated at 50 °C for 10 min. In the same way, the α-L-rhamnosidase activity was determined according to the above HPLC method.

**Fig. 1:**
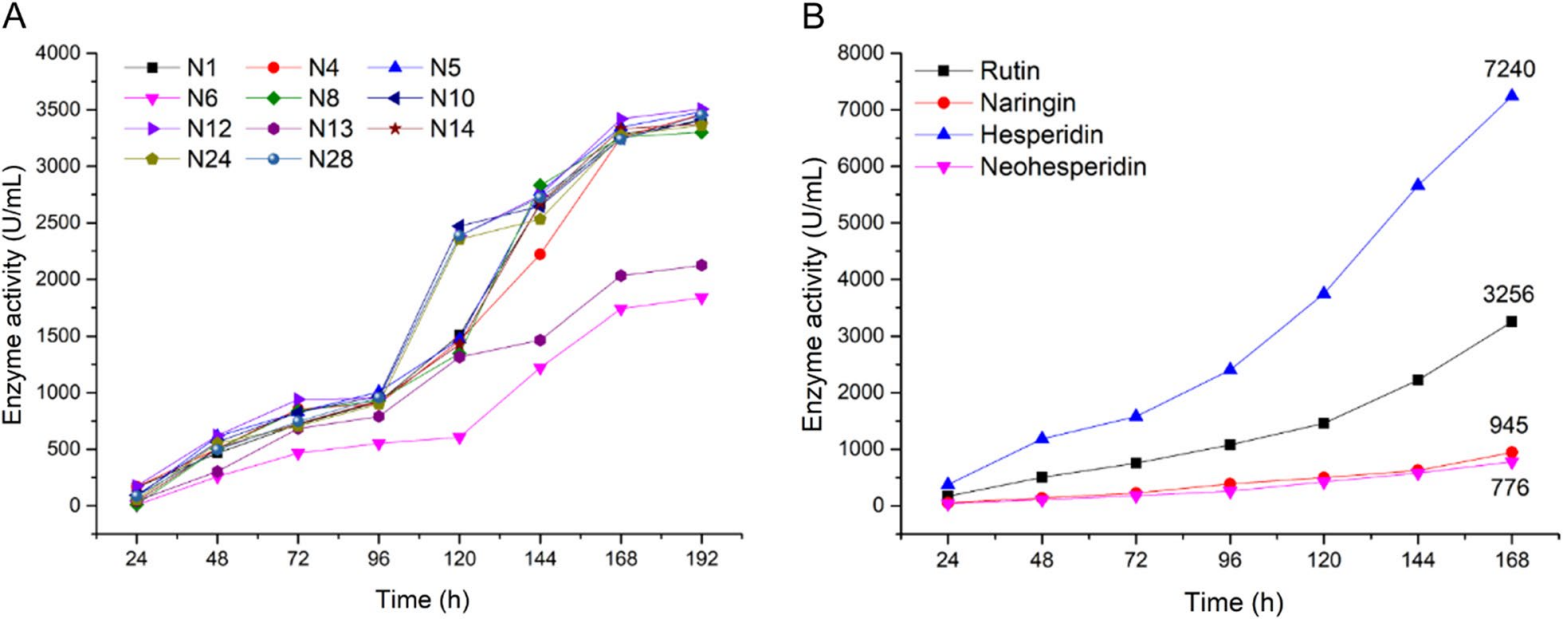
**A** Enzyme activity of α-L-rhamnosidase produced by 11 engineered strains (rutin as substrate). **B** The difference in enzyme activity of N12-Rha on different substrates.

### Preparation of α-L-rhamnosidase structure

The tertiary structure of α-L-rhamnosidase was modeled into PDB format *via* I-TASSER server (Roy et al. 2010). The energy of the 3D model was minimized through molecular mechanics method MM2. In order to evaluate the mode stability, we used Ramachandran plot analysis and Verify-3D server to score the PDB model.

### Quantum chemistry calculation of substrates

Here, the Gaussian09 program was used to perform quantum chemistry calculation. The initial geometric structures of hesperidin, naringin and myricetrin were obtained from the ChemSpider database (http://www.chemspider.com/). The semi-empirical algorithm AM1 was used to preliminarily optimize the substrate structure. The density functional algorithm B3LYP/6-311G(d) basis set was used for frequency analysis and structural optimization. The convergence criteria for geometric optimization were maximum force <0.000450, RMS force <0.000300, maximum displacement <0.001800 and RMS displacement <0.001200. The NBO net charge distribution, frontline molecular orbital energy, energy gap and ionization potential were analyzed (Boese and Martin 2004; Carpenter and Weinhold 1988).

### Molecular docking of protein-ligand binding

The interaction of α-L-rhamnosidase protein with hesperidin, naringin and myricetrin ligands was analyzed by AutoDock 4.2. The grid center was Asp256 αC (71.167, 71.322, 77.791) and the size of the grid box was (86 × 84 × 106). After 100 docking times, 50 ligand poses were evaluated and the optimal docking conformation was obtained (Saikia and Bordoloi 2019). The docking results were analyzed and plotted with PyMol software.

### Molecular dynamics simulation of the complexes of α-L-rhamnosidase and different substrates

The complex structure of α-L-rhamnosidase and different substrates was obtained from the optimal docking conformation produced by molecular docking, and Gromacs 2019.4 was used to perform MD simulation with Amber99SB-ILDN force field. The complex was solvated in a periodic cubic box with a distance of 1 nm between solute and the edge of the box, then the SPC water were added to the system. The counter ions (Na^+^, Cl^−^) were assigned with a concentration of 0.15 mol/L to neutralize the system. After the energy optimization, a constrained dynamics simulation was performed at 800 ps using the constrained dynamics method, and the temperature of the system was increased from 50 K to the 298 K required for the simulation by a step-up method. After that, the weak coupling method was used to keep the temperature and pressure (100 kPa) stable in the system, and the coupling time constants of temperature and pressure were both 0.1 ps. The long-range electrostatic interaction of the composite system was calculated using the particle mesh ewald (PME) method. The truncation radius of van der Waals and Coulomb interaction is 1.0 nm, and the short-range adjacent truncation distance is 1.0 nm. The simulation step size was 2 fs, and a 20 ns free kinetic simulation was performed for the three systems, and conformations were acquired every 2 ps (Pall et al. 2020; Rakhshani et al. 2019).

### Hydrogen bond and hydrophobicity analysis

Trajectories of MD simulations of α-L-rhamnosidase and substrate complex were sampled to analyze the hydrogen bond formation and hydrophobic interaction via LigPlot + 1.4.5 software (Laskowski and Swindells 2011).

### MM-PBSA Interaction Energy calculation

The g_mmpbsa tool was used to calculate the interaction energy (M-PBSA) of the substrates and α-L-rhamnosidase. The lower the interaction energy, the higher the affinity between the receptor and the ligand. Decomposing the total interaction energy into individual residues, the contribution of each residue can be shown (Genheden and Ryde 2015).

### Statistical analysis

One-way ANOVA and Duncan’s multiple range test were employed to study differences between the three groups. *P* < 0.05 were considered statistically significant. SPSS 22.0 software was used for experimental data processing.

## Results

### Subcloning, expression and purification of recombinant α-L-rhamnosidase

After the recombinant pPIC9K-α-L-rhamnosidase plasmid was transformed into *P. pastoris* GS115, positive clones were selected for PCR amplification of the target fragment to verify false positives. As shown in Additional file 1: Fig. S1A, the target gene fragment size is 1955 bp, and a total of 11 positive clones were screened. After SDS-PAGE analysis (Additional file 1: Fig. S1B), the 192 h fermentation supernatant was separated by Ni column affinity chromatography and digested with N-glycosidase F to obtain deglycosylated α-L-rhamnosidase.

### Identification for Engineered strains with high α-L-rhamnosidase activity

The 11 engineered strains (N1, N4, N5, N6, N8, N10, N12, N13, N14, N24 and N28) were cultured for 192 h. The expression of α-L-rhamnosidase was induced by adding methanol every 24 h, and the enzyme activity was measured with rutin as a substrate. The α-L-rhamnosidase activity curve of 11 strains (Fig. 1A) showed that the enzyme activity increased with the increase of culture time. The enzyme activity increased slowly in the first 4 days. From the 4th to the 7th day, the increase rate of the enzyme activity increased significantly. After the 7th day, the increase rate of the enzyme activity became flat again. Compared with other strains, the α-L-rhamnosidase produced by the strain N12 maintained the highest activity during the culture period, and the activity reached 3505.6 U/mL on the 8th day. Therefore, the strain N12 was confirmed as an engineered strain with high α-L-rhamnosidase activity.

**Fig. 2:**
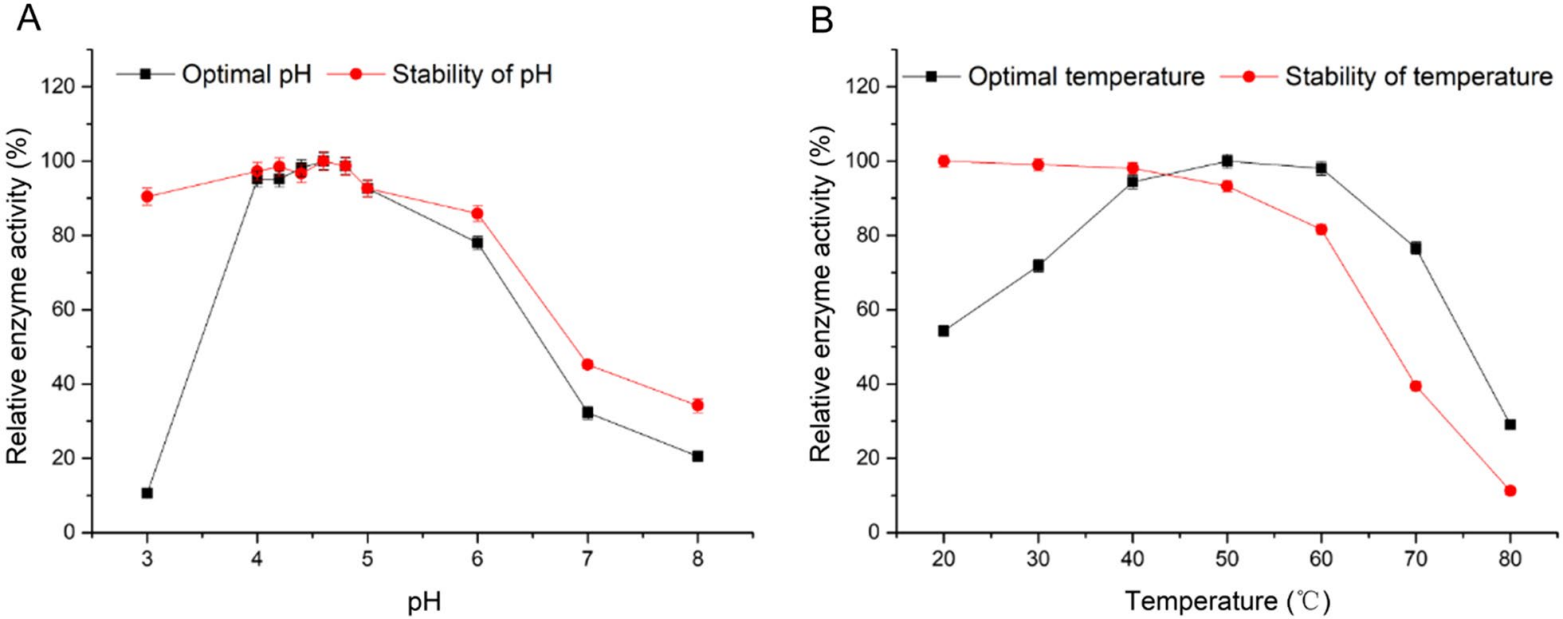
Effects of pH **A** and temperature **B** on recombinant α-L-rhamnosidase N12-Rha.

### Substrate specificity and affinity difference

Naringin, rutin, hesperidin, neohesperidin, and myricetin were used as substrates to determine the activity of the recombinant α-L-rhamnosidase N12-Rha produced by strain N12 on the 7th day. As shown in Table 1, It is shown that the recombinant α-L-rhamnosidase N12-Rha can hydrolyze rutin, naringin, hesperidin, neohesperidin, but not myricetin. According to the structural analysis of five flavonoids, naringin and neohesperidin contain α-1,2 glycosidic bond, hesperidin and rutin contain α-1,6 glycosidic bond, and myricetin contains α-1,3 glycosidic bond. Therefore, the substrate specificity of the recombinant α-L-rhamnosidase N12-Rha is that it can hydrolyze the flavonoids containing α-1,2, α-1,6 glycosidic bonds, but cannot hydrolyze the C-O bond directly connected to the heterocycle.

**Table 1:** Substrate specificity of recombinant α-L-rhamnosidase N12-Rha

Substrate	Type of linkage	Activity (%)
Naringin	α–1,2	100
Neohesperidin	α–1,2	82.12
Myricetrin	α–1,3	0
Hesperidin	α–1,6	766.14
Rutin	α–1,6	344.55

To further compare and analyze the activity changes of the recombinant α-L-rhamnosidase N12-Rha on four specific substrates (naringin, rutin, hesperidin and neohesperidin) during fermentation. As shown in Fig. 1B, during the seven days of fermentation, the activity of N12-Rha increased over time, and the affinity for the four substrates was ranked from strong to weak: hesperidin > rutin > naringin > neohesperidin. The affinity of the recombinant α-L-rhamnosidase N12-Rha to hesperidin and rutin containing α-1,6 glycosidic bond were significantly greater than that of naringin and neohesperidin containing α-1,2 glycosidic bond. This result indicates that the hydrolysis activity of N12-Rha to α-1,6 glycosidic bond was significantly higher than that of α-1,2 glycosidic bond.

### pH and the temperature stability

Figure 2 shows the changes of the recombinant α-L-rhamnosidase N12-Rha activity at different pH ​​and temperatures. It can be seen from Fig. 2A that the optimal pH of N12-Rha reaction is 4.6, and the N12-Rha activity remains above 85% at pH 4–5, however, the enzyme activity is lower than 25% at pH 3 and 8. The results indicate that the recombinant α-L-rhamnosidase N12-Rha is a weakly acidic enzyme, and it is not suitable to react under strong acid and strong base conditions. when the recombinant α-L-rhamnosidase N12-Rha was treated in pH 3–6 at 4 ℃ for 24 h, the activity remained above 80%. The results indicate that N12-Rha has a certain tolerance to acidic environments.

**Fig. 3:**
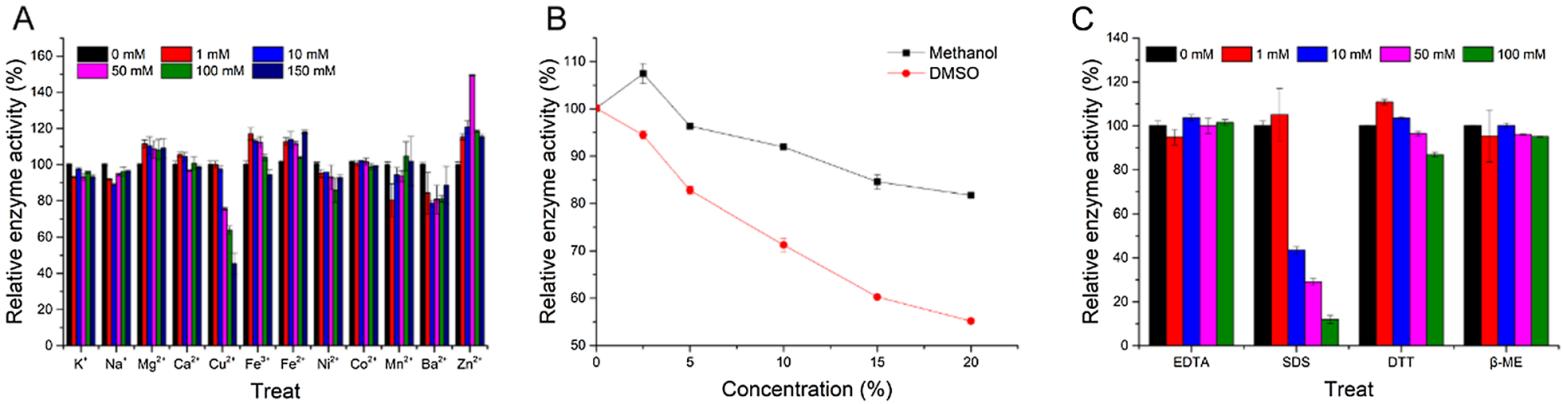
Effects of metal ions (**A**), organic solvents **B** and other effectors **C** on recombinant α-L-rhamnosidase N12-Rha

Figure 2B shows that the optimal reaction temperature of the recombinant α-L-rhamnosidase N12-Rha is 50 °C, and within the reaction temperature range of 40–60 ℃, the N12-Rha activity remains above 85%, this indicates that N12-Rha is a relatively heat-resistant enzyme. The analysis of the temperature tolerance found that the recombinant α-L-rhamnosidase N12-Rha activity decreased with increasing temperature. When N12-Rha was treated at 20–60 ℃ for 2 h, the activity remained above 80%. But when the temperature exceeds 60 ℃ the N12-Rha activity decreases rapidly. To sum up, the results show that the optimal reaction pH is 4.6 and the optimal reaction temperature of N12-Rha is 50 ℃.

### Effects of metal ions, organic solvents and other effectors

As we all know, the catalytic activity of enzymes will be affected by the metal ions, organic solvents and some effectors in the system. Figure 3A shows that K^+^, Na^+^, Ca^2+^, Ni^2+^, Co^2+^, and Mn^2+^ at the concentrations of 1 mM, 10 mM, 50 mM, 100 mM and 150 mM have little effect on the enzyme activity of N12-Rha. However, the N12-Rha activity is increased by adding 1 mM, 10 mM, 50 mM, 100 mM and 150 mM Mg^2+^, Fe^3+^, Fe^2+^, and Zn^2+^. Interestingly, when 50 mM Zn^2+^ is added, the relative enzyme activity of N12-Rha is 149.51%, and the enzyme activity reaches the highest. This result indicates that the hydrolysis process of naringin by N12-Rha requires a certain concentration of Zn^2+^. Ba^2+^ at the concentrations of 1 mM, 10 mM, 50 mM, 100 mM and 150 mM has a certain inhibitory effect on the enzyme activity of N12-Rha. 1 mM and 10 mM Cu^2+^ have no significant effect on the enzyme activity of N12-Rha, but when the concentration of Cu^2+^ is higher than 10 mM, the enzyme activity of N12-Rha decreases as the concentration of Cu^2+^ increases. The relative enzyme activity of N12-Rha treated with 150 mM Cu^2+^ is 45.37%, the enzyme activity is minimized.

Figure 3B shows the enzyme activity change of N12-Rha in the presence of organic solvents (methanol and DMSO). When the methanol concentration is in the range of 0–20% (v/v), the relative activity of N12-Rha remains above 80%, and 2.5% (v/v) DMSO solution has a certain promotion effect on the relative enzyme activity. When the DMSO concentration is in the range of 0–20% (v/v), the relative activity of N12-Rha remains above 50%. In summary, N12-Rha shows better tolerance to organic solvents.

The results showed that EDTA and β-ME at concentrations of 1 mM, 10 mM, 50 mM and 100 mM had almost no effect on the enzyme activity of N12-Rha (Fig. 3C). Only the high concentration of 100 mM DTT reduced the relative enzyme activity to 86.83%. 10 mM, 50 mM and 100 mM SDS reduced the relative enzyme activity to 43.54%, 29.08% and 12.04%, respectively. In summary, the presence of SDS can significantly inhibit the activity of N12-Rha.

### Analysis of the substrate structure-specificity relationship through quantum chemistry calculation

**Fig. 4:**
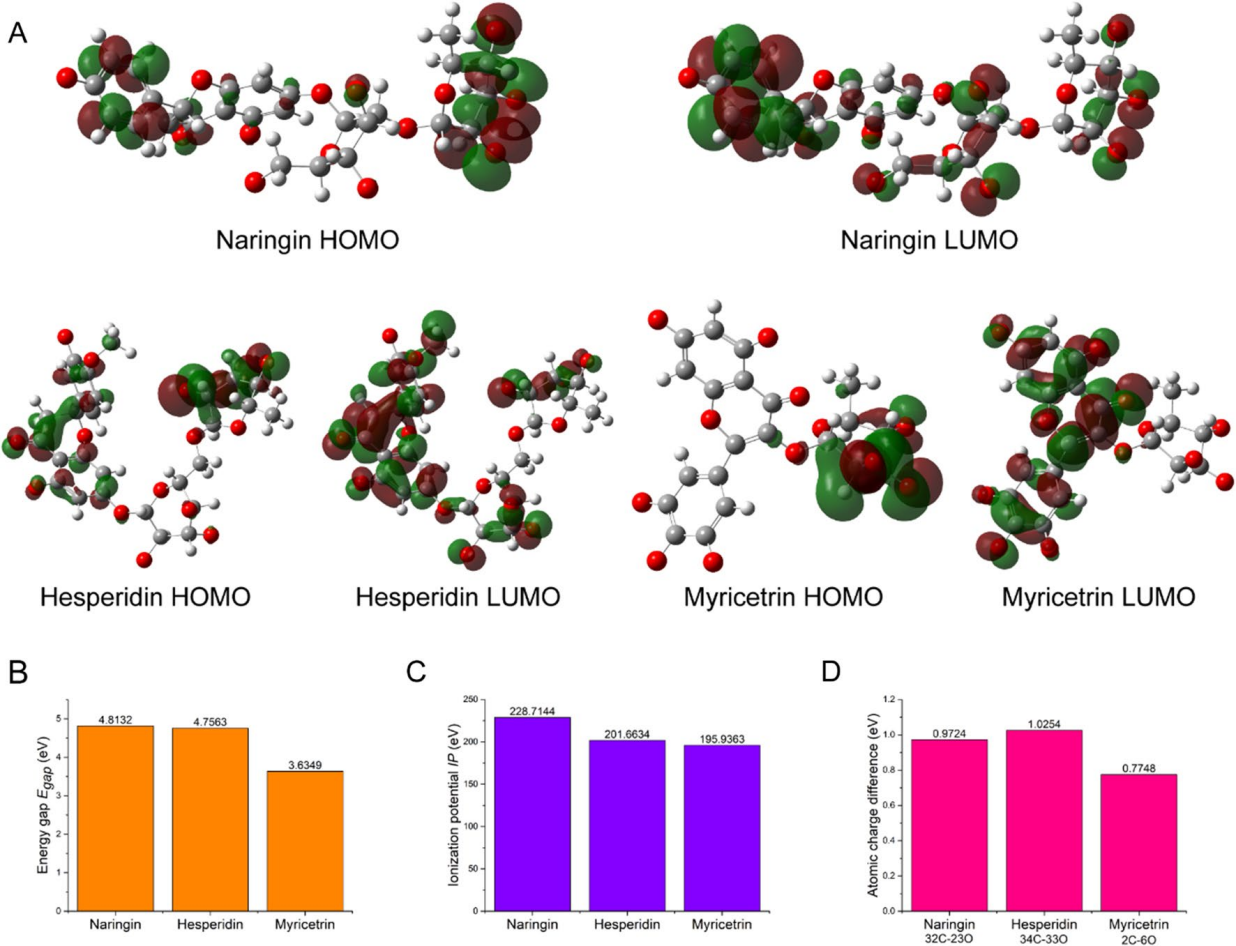
**A** HOMO and LUMO distribution of protonated substrates. Red: positive, Green: negative. Energy gap **B** and ionization potential **C** of protonated substrates. **D** The C-O charge difference of the three substrate-specific glycosidic bonds

Hesperidin, naringin and myricetrin are used as substrate models for molecular simulation studies because of their differences in glycosidic bond types. The L-rhamnose of hesperidin is connected by α-1,6 glycosidic bond, the L-rhamnose of naringin is connected by α-1,2 glycosidic bond, and the L-rhamnose of myricetrin is directly connected to the heterocyclic ring by α-1,3 glycosidic bond. The structures of hesperidin, naringin and myricetrin were optimized by quantum chemistry calculation methods to obtain a reasonable substrate structure. Molecular orbital theory indicates that the distribution of the highest occupied molecular orbital (HOMO) and the lowest unoccupied molecular orbital (LUMO) plays a key role in the occurrence of molecular reactions (Solov’ev et al. 2018). Figure 4A shows the distribution of the frontier molecular orbitals of the three substrates. The HOMOs and LUMOs of protonated hesperidin and naringin are distributed on their L-rhamnose groups, indicating that the electrons on the L-rhamnose are easier to transfer. The LUMO is not distributed on the L-rhamnose group of protonated myricetrin, indicating that this group is not easy to accept electrons. The energy gap E_gap_ reflects the difficulty of electron transition. The lower the value of E_gap_, the less likely it is for electrons to transition (Balasubramani et al. 2018). It can be seen from Fig. 4B that the energy gap E_gap_ of myricetrin is significantly smaller than hesperidin and naringin, which shows that myricetrin is more stable than hesperidin and naringin.

Ionization potential (IP) is a parameter that characterizes the ability of molecules to obtain electrons. Figure 4C shows that the ionization potentials of hesperidin and naringin are significantly smaller than myricetrin, indicating that hesperidin and naringin are easier to obtain electrons. Molecular structure theory indicates that the greater the difference in charge between atoms, the easier it is for electrons to undergo transitions (Li et al. 2014). Figure 4D shows the C-O charge difference of the three substrate-specific glycosidic bonds. The C-O charge difference between the α-1,6 glycosidic bond of hesperidin is the largest, and the C-O charge difference of the α-1,3 glycosidic bond of myricetrin is the smallest. This indicates that the α-1,6 glycosidic bond of hesperidin is the easiest to break, while the C-O bond of the α-1,3 glycosidic bond of myricetrin is the most stable.

### MD trajectories analysis of α-L-rhamnosidase and substrates

After the structure of N12-Rha was optimized, a total of 93.13% of the dihedral angles of ϕ and ψ were within the allowable distribution, which indicates that the model is a normally distribute and has high reliability (Additional file 1: Fig. S2A). The 3D-1D scoring function was used to evaluate the match between the 3D configuration of N12-Rha and the amino acid sequence. Of the residues, 85.35% had an average 3D-1D score greater than 0.2, indicating that the construction of the 3D model of N12-Rha was reasonable (Additional file 1: Fig. S2B).

**Fig. 5:**
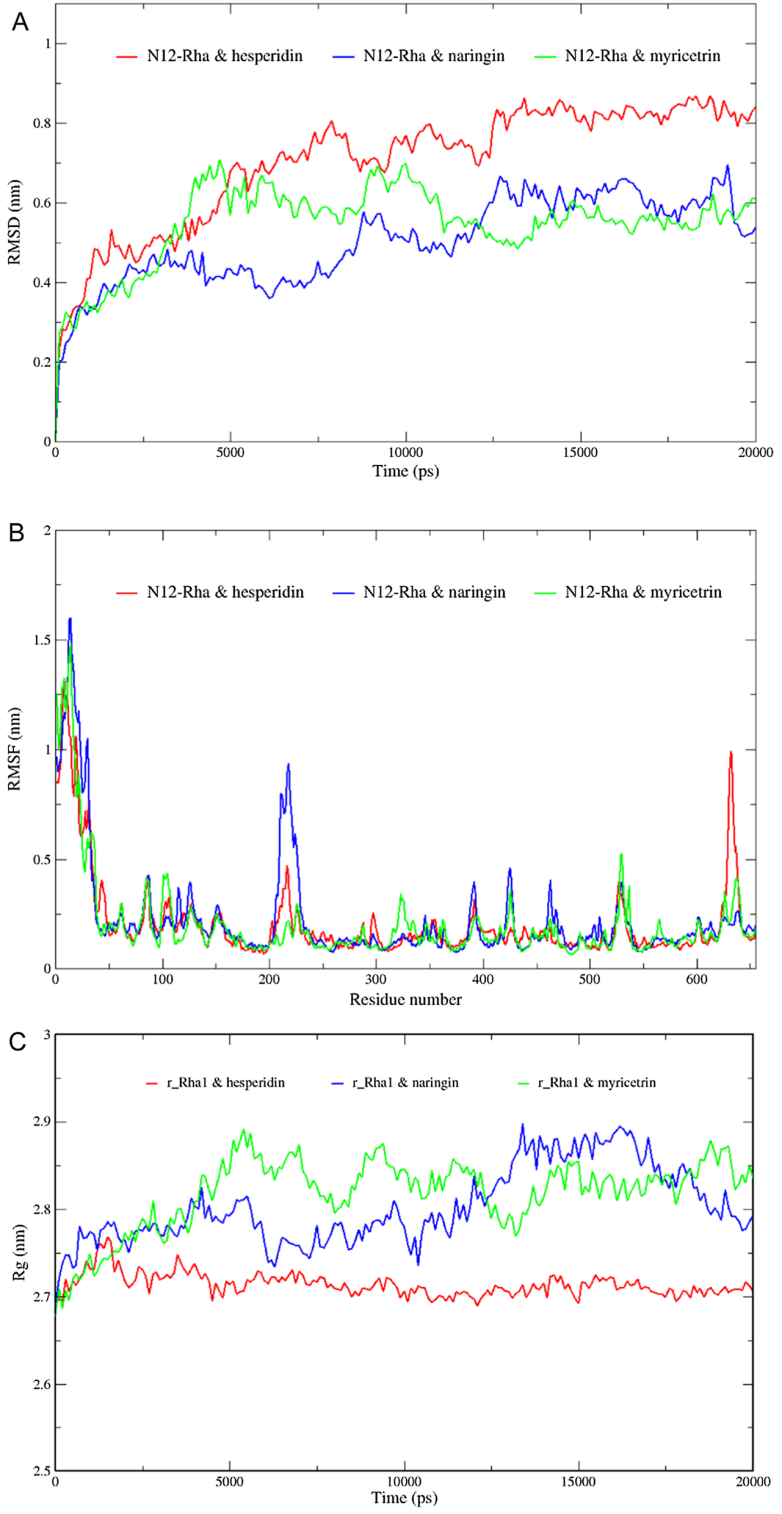
The RMSD (**A**), RMSF **B** and Rg **C** of N12-Rha binds to hesperidin, naringin and myricetrin in MD simulations

We performed 20 ns MD simulations on the molecular docking complexes of N12-Rha with hesperidin, naringin and myricetrin, respectively. The root mean square deviation (RMSD) characterizes the average distance between the current structure and the reference structure. If the RMSD changes little over time, it indicates that the system has reached a local equilibrium (Sargsyan et al. 2017). Figure 5A shows the results of the RMSD change with time during the 20 ns simulation. N12-Rha with hesperidin, naringin and myricetrin reached local equilibrium at about 15 ns. In the local equilibrium, N12-Rha with hesperidin has the largest RMSD, while N12-Rha with myricetrin has the smallest RMSD. The root mean square fluctuation (RMSF) characterizes the flexibility of protein molecules and the dynamic difference of each residue (Daghestani et al. 2019). The overall flexibility of N12-Rha with hesperidin and naringin is slightly higher than that of N12-Rha with myricetrin, Especially the changes in residues 200–250 are particularly obvious (Fig. 5B). The radius of gyration (Rg) characterizes the overall size of the protein in the dynamic system (Singh et al. 2018). The Rg of N12-Rha in the hesperidin system is significantly lower than that of the other two systems (Fig. 5C), indicating that other structures have become relatively looser.

### Analysis of hydrophobic interaction and hydrogen bonding

**Fig. 6:**
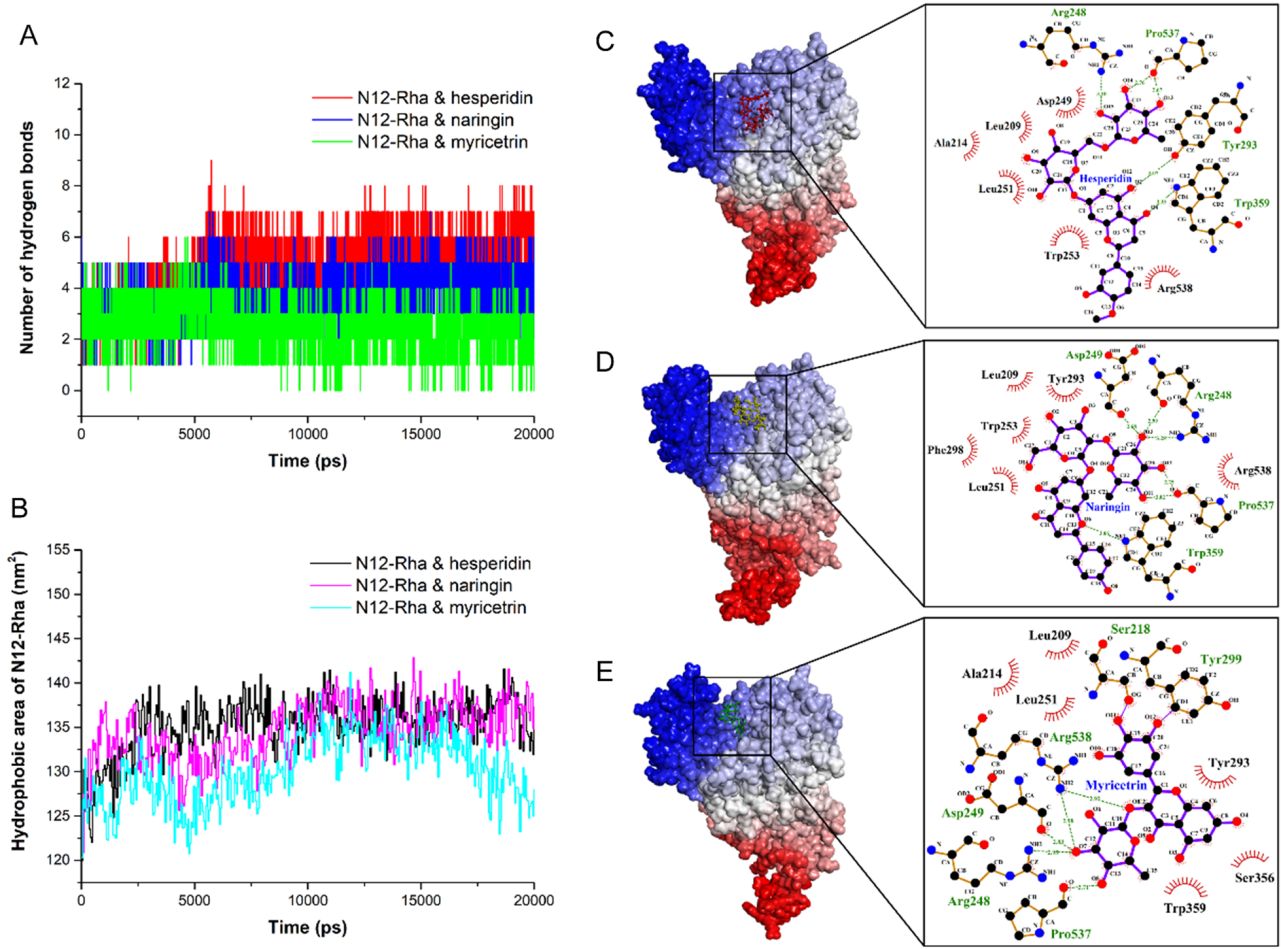
The hydrogen bond analysis **A** and hydrophobic area analysis **B** during 20 ns MD simulation, hydrophobic interaction and hydrogen bonding of hesperidin (**C**), naringin **D** and myricetrin **E** binding to N12-Rha during 15–20 ns MD simulation

During the MD simulation, the average number of hydrogen bonds between N12-Rha and hesperidin, and between N12-Rha and naringin gradually increased, while the number of hydrogen bonds between N12-Rha and myricetrin gradually decreased after 5 ns (Fig. 6A). The average number of hydrogen bonds of the N12-Rha with hesperidin, naringin and myricetrin were 4.9, 3.7 and 2.5, respectively. These results indicated that N12-Rha could have strong hydrogen bonding interaction with hesperidin and naringin, while the interaction between N12-Rha and myricetrin was weaker. In addition, the non-polar energy has a positive effect on the interaction energy(Kumari et al. 2014). Figure 6B shows the hydrophobic areas of N12-Rha interacting with hesperidin, naringin and myricetrin during the MD simulation, respectively. The average hydrophobic areas of the N12-Rha with hesperidin, naringin and myricetrin were 135.2 nm^2^, 134.5 nm^2^ and 130.2 nm^2^, respectively. Therefore, it is shown that N12-Rha has the strongest hydrophobic interaction with hesperidin and the weakest hydrophobic interaction with myricetrin.

Previously, the RMSD showed that the MD system reached local equilibrium at 15–20 ns. Table 2 shows the hydrogen bond occupancy during 15–20 ns MD simulation. Among them, Trp359 and Pro537 showed high hydrogen bond occupancy. Thus, Trp359 and Pro537 are key residues for the hydrogen bonding interaction of N12-Rha with the substrate. Meanwhile, Fig. 6C–E show the formation of hydrophobic interactions and hydrogen bonds of average structures during 15–20 ns MD simulation. It can be seen from Fig. 6C that when hesperidin binds to N12-Rha, Leu209, Ala214, Asp249, Leu251, Trp253 and Arg538 form hydrophobic interactions with hesperidin. Arg248, Tyr293, Trp359 and Pro537 form hydrogen bond interactions with hesperidin. Figure 6D shows that Leu209, Leu251, Trp253, Tyr293, Phe298 and Arg538 form hydrophobic interactions with naringin. Arg248, Asp249, Trp359 and Pro537 form hydrogen bond interactions with naringin. Figure 6E shows that Leu209, Ala214, Leu251, Tyr293, Ser356, Trp359, all form hydrophobic interactions with myricetrin. Arg248, Asp249, Pro537 and Arg538 form hydrogen bond interactions with myricetrin.

**Table 2:** Hydrogen bond occupancy during 15–20 ns MD simulation

Complexes	Donor	Acceptor	Occupancy (%)
N12–Rha & Hesperidin	Hesperidin O15	Arg248 NH2	12.0
	Hesperidin O2	Tyr293 OH	24.2
	Hesperidin O4	Trp359 NE2	41.3
	Hesperidin O13	Pro537 O	56.9
N12–Rha & Naringin	Naringin O13	Arg248 O	15.7
	Naringin O13	Asp249 O	11.8
	Naringin O6	Trp359 NE1	47.8
	Naringin O12	Pro537 O	31.4
N12–Rha & Myricetrin	Myricetrin O7	Arg248 NH2	10.1
	Myricetrin O7	Asp249 O	8.5
	Myricetrin O8	Pro537 O	43.4
	Myricetrin O5	Arg538 NH2	11.6

### MM-PBSA interaction energy between α-L-rhamnosidase and substrate

Molecular mechanics Poisson-Boltzmann surface area (MM-PBSA) is a method used to calculate the interaction energy after the molecular dynamics of the receptor and the ligand. The calculated interaction energy can be used to reflect the ligand and the receptor combined stability (Westermaier et al. 2017). Table 3 lists the interaction energy between N12-Rha and hesperidin, naringin and myricetrin and the energy value of each component. This indicates that the affinity of N12-Rha for the three substrates is ranked from high to low: hesperidin > naringin > myricetrin, which is consistent with the above experimental results. The interaction between N12-Rha and hesperidin and naringin is dominated by van der Waals forces. Coulomb electrostatic interaction and non-polar solvation energy contribute little to the interaction energy.

**Table 3:** Interaction energy of the N12-Rha with hesperidin, naringin and myricetrin based on MM-PBSA analysis, respectively

The interaction energy (kJ/mol)	Complexes		
	N12–Rha & hesperidin	N12–Rha & naringin	N12–Rha & myricetrin
Van der Waal energy	− 118.712 ± 1.244	− 93.232 ± 1.521	− 86.340 ± 1.332
Electrostatic energy	− 33.498 ± 0.431	− 23.811 ± 0.220	− 73.750 ± 0.402
Polar solvation energy	118.870 ± 1.906	86.788 ± 1.737	140.665 ± 1.834
Non–Polar solvation energy	− 18.182 ± 0.371	− 17.071 ± 0.557	− 17.644 ± 0.331
Total energy (MM–PBSA)	− 51.522 ± 0.526	− 47.326 ± 0.637	− 37.068 ± 0.855

**Fig. 7:**
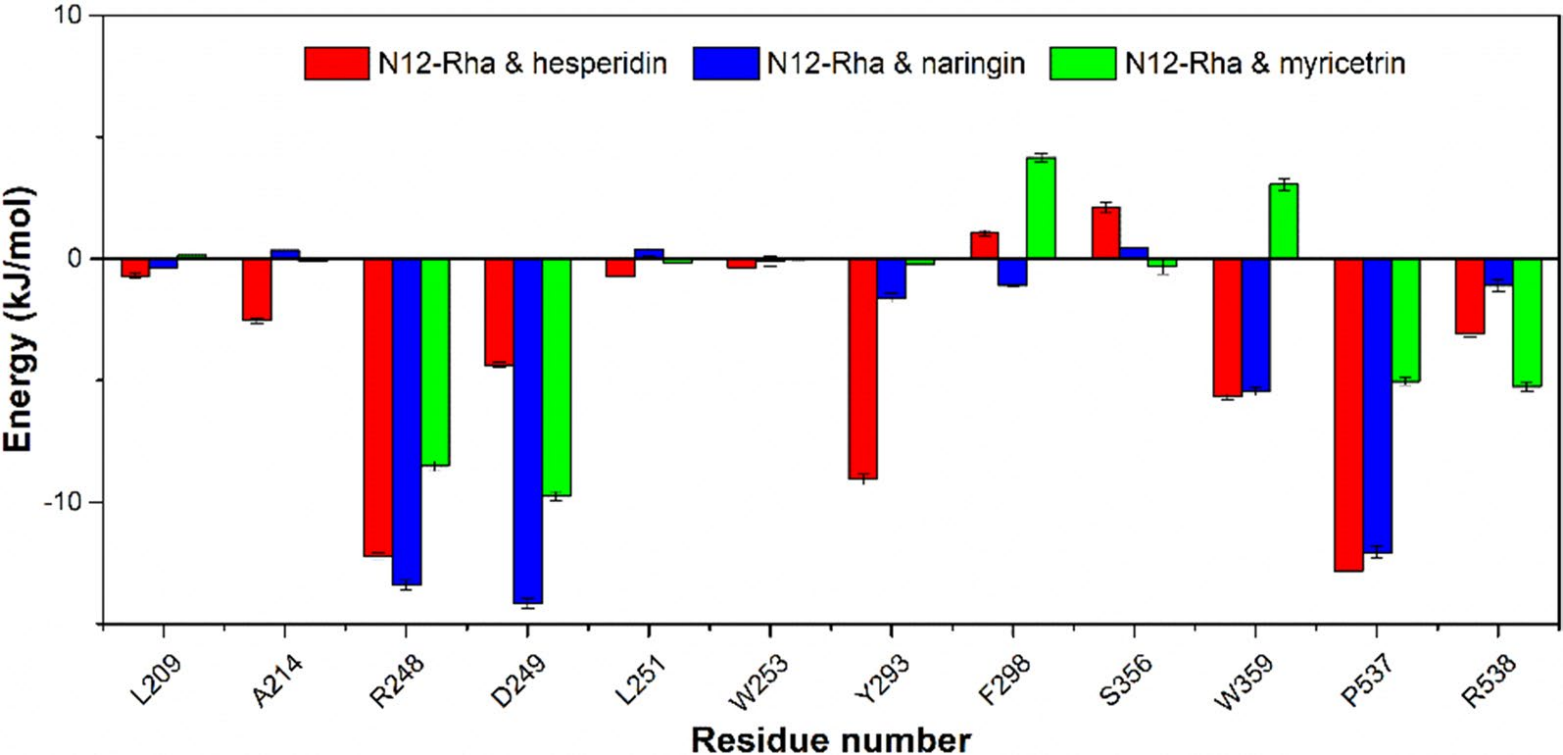
Interaction energies of the hesperidin and naringin, myricetrin with N12-Rha ((α/α)_6_-barrel domain) based on MM-PBSA analysis during 15–20 ns MD simulation

Obviously, the key residues that have a greater contribution to hydrogen bond formation and hydrophobic interactions are concentrated in the (α/α)_6_-barrel domain (Glu183-Thr557) of N12-Rha, which indicates that the (α/α)_6_-barrel domain is the catalytic domain of N12-Rha. Figure 7 shows the residues in the (α/α)_6_-barrel domain of N12-Rha that contribute significantly to the total interaction energy. Arg248, Asp249, Tyr293, Pro537 and Arg538 make positive contributions to the interaction of N12-Rha to the three substrates. Meanwhile, Trp359 make a positive contribution to the interaction of N12-Rha to hesperidin and naringin, while making a negative contribution to the interaction between N12-Rha and myricetrin.

## Discussion

α-L-Rhamnosidase can specifically catalyze the hydrolysis of substances ending with α-L-rhamnose, such as hesperidin, rutin and naringin, to produce L-rhamnose. It can remove the bitter taste of naringin in the juice, clear the turbidity of the beverage caused by hesperidin crystals, and can hydrolyze to produce many prodrugs, such as Prunin (Li et al. 2018). Therefore, α-L-rhamnosidase is an important hydrolase in food processing and pharmaceuticals. The α-L-rhamnosidase produced by *Aspergillus niger* JMU-TS528 also has these catalytic properties. However, due to the complex metabolites, the production of α-L-rhamnosidase by natural *Aspergillus niger* has high purification cost and low recovery rate. In addition, its enzyme activity is low, about 88.9 U/mL (for naringin) (Li et al. 2016). In this study, we optimized and synthesized the α-L-rhamnosidase gene sequence of *Aspergillus niger* JMU-TS528 by targeting the *P. pastoris* GS115 expression system, recombined it into the pPIC9K vector and transferred it into *P. pastoris* GS115. A recombinant engineered strain N12 with high enzyme activity was selected. The recombinant α-L-rhamnosidase N12-Rha can specifically hydrolyze hesperidin, rutin, naringin and neohesperidin, but not myricetrin.

Li et al. used the total RNA of wild *Aspergillus niger* JMU-TS528 as a template to obtain α-L-rhamnosidase cDNA and ransferred into *P. pastoris* GS115. The catalytic activity of recombinant α-L-rhamnosidase r-Rha1 was 1118.5 U/mL for hesperidin, 1055.2 U/mL for rutin, and 771.9 U/mL for naringin (Li et al. 2016). In this study, The catalytic activity of the recombinant α-L-rhamnosidase N12-Rha was 7240 U/mL for hesperidin, 3256 U/mL for rutin, and 945 U/mL for naringin (Fig. 1B). N12-Rha and r-Rha1 all show higher catalytic activity on α-1,6 glycosidic bond than α-1,2 glycosidic bond. Compared with r-Rha1, N12-Rha increased the activity of naringin containing α-1,2 glycosidic bond by 22.4%. The activity of N12-Rha on rutin containing α-1,6 glycosidic bonds is 3.09 times that of r-Rha, and the activity of N12-Rha on hesperidin containing α-1,6 glycosidic bonds is 6.47 times that of r-Rha. The enzyme activity of N12-Rha is 10.63 times that of wild *Aspergillus niger* JMU-TS528 α-L-rhamnosidase (for naringin) (Li et al. 2016). Although the amino acid sequence of N12-Rha and r-Rha1 are the same, the N12-Rha gene sequence is more suitable for Pichia expression. Therefore, the different post-translational modification of yeast may lead to differences in the properties of its partial enzymes. To the best of our knowledge, this is the first report on the highest activity of heterologous expression of *Aspergillus* α-L-rhamnosidase.

The optimal pH of N12-Rha is 4.6 and the optimal temperature is 50℃ (Fig. 2), which is consistent with the reported optimal pH range (4-6.5) and the optimal temperature range (40–60℃) of *Aspergillus* α-L-rhamnosidase. Gerstorferová et al. expressed *Aspergillus terreus* α-L-rhamnosidase in *P. pastoris* KM71H with an optimal pH of 4.0 and an optimal temperature of 60 °C (Gerstorferová et al. 2012). Similarly, Liu et al. expressed the α-L-rhamnosidase of *Aspergillus niger* DLFCC-90 in *P. pastoris* GS115, with an optimal pH of 5.0 and an optimal temperature of 50 °C (Liu et al. 2012). N12-Rha can tolerate the acidic environment of pH 3–6 and maintain more than 80% enzyme activity (Fig. 2A), so it is suitable for the production process of fruit juice or wine making. In addition, N12-Rha can also tolerate most metal ions within a concentration of 100 mM (Fig. 3A). Li et al. reported that r-Rha1 was strongly inhibited by iron ions (including Fe^3+^ and Fe^2+^). However, the N12-Rha activity is increased by adding 1 mM, 10 mM, 50 mM, 100 mM and 150 mM Fe^3+^, Fe^2+^ And Cu^2+^ at a concentration higher than 10 mM can significantly inhibit the activity of N12-Rha. Interestingly, 50 mM Zn^2+^ can significantly enhance the enzymatic activity of N12-Rha, but if it is too high, it will inhibit it. So far, the promotion and inhibition mechanisms of zinc ions, copper ions and iron ions on N12-Rha activity are still unclear(Baudrexl et al. 2019). In addition, N12-Rha can tolerate EDTA, DTT and β-ME within 100 mM, and maintain more than 80% activity in 20% (v/v) methanol, and maintain more than 50% activity in 20% (v/v) DMSO (Fig. 3B, C). these characteristics make N12-Rha suitable for more applications.

According to reports, most α-L-rhamnosidases have higher enzymatic activity on α-1,2 glycosidic bond than on α-1,6 glycosidic bond(Yadav et al. 2010). Interestingly, N12-Rha has a stronger substrate affinity for α-1,6 glycosidic bond than α-1,2 glycosidic bond and its hydrolysis activity on α-1,6 glycosidic bond is significantly higher, but little catalytic activity for myricetein containing α-1,3 glycosidic bond. The difference in the affinity of N12-Rha substrates prompted us to further explore its molecular mechanism. The quantum chemistry calculations and MD simulations were used to explain the differences in the affinity of N12-Rha to substrates from molecular modelling. First, the frontier molecular orbitals of the protonated hesperidin and naringin are distributed on the L-rhamnose group, but the LUMO of myricetrin is not distributed on the L-rhamnose group (Fig. 4A), which shows that hesperidin and naringin L-rhamnose groups are more likely to react than myricetrin L-rhamnose group. Secondly, the energy gap and ionization potential of the molecules indicate that hesperidin and naringin are more prone to electronic transitions than myricetrin at the overall molecular level, thereby causing molecular reactions (Fig. 4B, C). Finally, we compared the atomic charge differences of the C-O bonds of α-1,2, α-1,3 and α-1,6 of the three substrates (Fig. 4D). In comparison, the α-1,6 bond is the easiest to break, and the α-1,2 bond is second, and the α-1,3 bond is the strongest. The charge difference of the α-1,2, α-1,3 and α-1,6 glycosidic bonds of different substrates reflect the difficulty of hydrolysis of the C-O bond. The above quantum chemistry parameters are consistent with the results of the difference in substrate affinity, which shows that quantum chemistry indicators can be used for the research of α-L-rhamnosidase substrate specificity. In addition, the free energy of the transition state of the reaction of N12-Rha with the substrate can be further calculated using the QM/MM method in the future.

MD simulation can study the dynamic process of biomolecule interaction (Daghestani et al. 2019). We performed MD simulations on the complexes of N12-Rha docked with hesperidin, naringin and myricetrin to reveal the interaction between N12-Rha and the substrate. The hydrophobic interaction between N12-Rha and the substrate and the formation of hydrogen bonds were further analyzed during 15–20 ns local equilibrium (Fig. 6; Table 2), we found that Arg248, Asp249, Tyr293, Pro537 and Arg538 make positive contributions to the interaction of the substrates to N12-Rha. Trp359 can form stable hydrogen bonds with hesperidin and naringin during 15–20 ns local equilibrium, but not with myricetrin. Meanwhile, In the systems where hesperidin and naringin are located, Trp359 shows positive interaction energy, while in the system where myricetrin is located, it shows negative interaction energy (Fig. 7). It is speculated that Trp359 may be the key residue that catalytic activity of N12-Rha on α-1,6 glycosidic bond is higher than α-1,2 glycosidic bond. In addition, the MD results show that van der Waals force makes the main contribution for the interaction energy of hesperidin, naringin and N12-Rha. The coulomb electrostatic force and non-polar solvation energy have little effect on the interaction. According to the contribution of each residue in the interaction energy (Table 3; Fig. 7), we found that the main catalytic sites of N12-Rha binding to the substrate are located in the (α/α)_6_-barrel domain.

In conclusion, the recombinant α-L-rhamnosidase N12-Rha was codon-optimized and expressed extracellularly in *P. pastoris* GS115 and its activity was 10.63 times that of native α-L-rhamnosidase from *Aspergillus niger* JMU-TS528. Quantum chemistry calculation methods revealed that the charge difference of α-1,2, α-1,3 and α-1,6 glycosidic bonds determines the difficulty of the substrates being catalyzed from molecular modelling. Molecular dynamics simulations showed the core catalytic domain of N12-Rha and identified key residue Trp359 that may affect substrate specificity. This work not only enhanced the affinity of α-L-rhamnosidase N12-Rha through heterologous expression, but also revealed the interactions between N12-Rha and the substrates using molecular modelling.

## Supplementary Information

**Additional file 1: Fig.S1.** **A** PCR amplification of α-L-rhamnosidase gene from 11 positive clones (N1, N4, N5, N6, N8, N10, N12, N13,N14, N24 and N28). **B** SDS-PAGE analysis of N-glycosidase F treated N12-Rha.Lane 1, untreated N12-Rha; lane 2, N12-Rha treated with N-Glycosidase F. **Fig.S2.** Ramachandran plot **A** and Verify-3D **B** of N12-Rha model

## Data Availability

All data generated or analyzed during this study are included in this published article (and its supplementary information files).
