# Supplementary material for: Insights into glycosidic bond specificity of an engineered selective α-L-rhamnosidase N12-Rha via activity assays and molecular modelling
Source: AMB Express. 2022 Nov 12;12:143. doi: 10.1186/s13568-022-01489-5 (PMC9653530; doi:10.1186/s13568-022-01489-5)
Supplement: Supplementary file 1 — Additional file 1: Fig.S1. A PCR amplification of α-L-rhamnosidase gene from 11 positive clones (N1, N4, N5, N6, N8, N10, N12, N13,N14, N24 and N28). B SDS-PAGE analysis of N-glycosidase F treated N12-Rha.Lane 1, untreated N12-Rha; lane 2, N12-Rha treated with N-Glycosidase F. Fig.S2. Ramachandran plot A and Verify-3D B of N12-Rha model [file 13568_2022_1489_MOESM1_ESM.docx]

**Additional file 1**

**Insights into Glycosidic Bond Specificity of an Engineered Selective α-L-rhamnosidase N12-Rha via Activity Assays and Molecular Modelling**

Bo Yu^a^, Shiyu Luo^b^, Yuhan Ding^c^, Zijie Gong^b^, Ting Nie^a, d *^

^a^ *Jiangxi-OAI Joint Research Institute, Nanchang University, Nanchang, Jiangxi Province 330047, China*

^b^ *College of Chemistry, Nanchang University, Nanchang, Jiangxi Province 330031, China*

^c^ *Medical College of Dalian University, Dalian, Liaoning Province 116622, China*

^d^ *State Key Laboratory of Microbial Metabolism, Joint International Research Laboratory of Metabolic & Developmental Sciences, School of Life Sciences and Biotechnology, Shanghai Jiao Tong University, Shanghai 200240, China*

^*^Corresponding Author: Ting Nie

Email: [nieting@sjtu.edu.cn](mailto:nieting@sjtu.edu.cn)

Fig. S1. (A) PCR amplification of α-L-rhamnosidase gene from 11 positive clones (N1, N4, N5, N6, N8, N10, N12, N13, N14, N24 and N28). (B) SDS-PAGE analysis of N-glycosidase F treated N12-Rha. Lane 1, untreated N12-Rha; lane 2, N12-Rha treated with N-Glycosidase F.

Fig. S2. Ramachandran plot (A) and Verify-3D (B) of N12-Rha model.

Fig. S1.


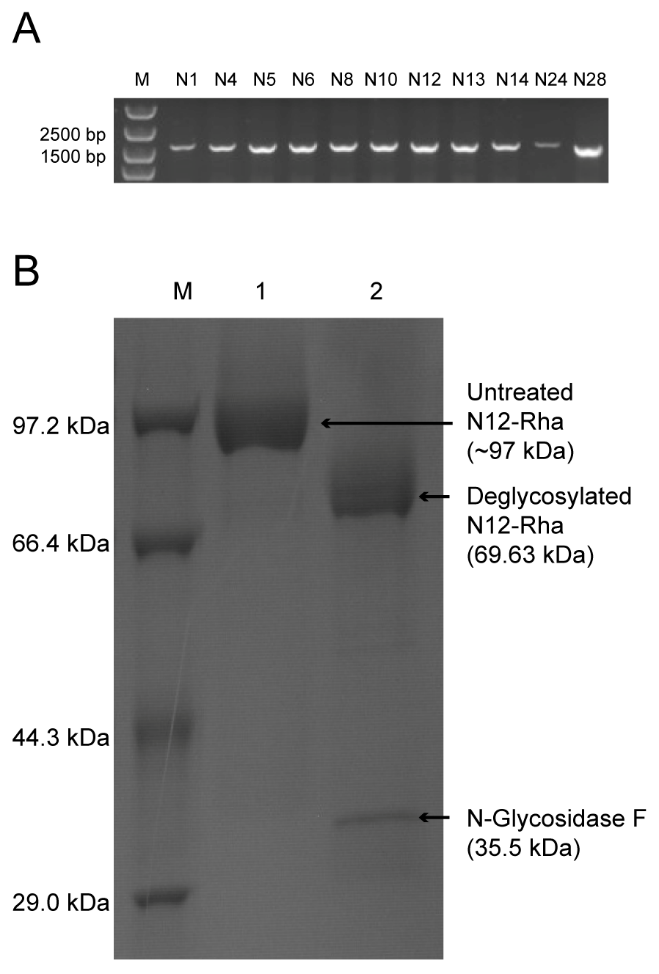


Fig. S2.


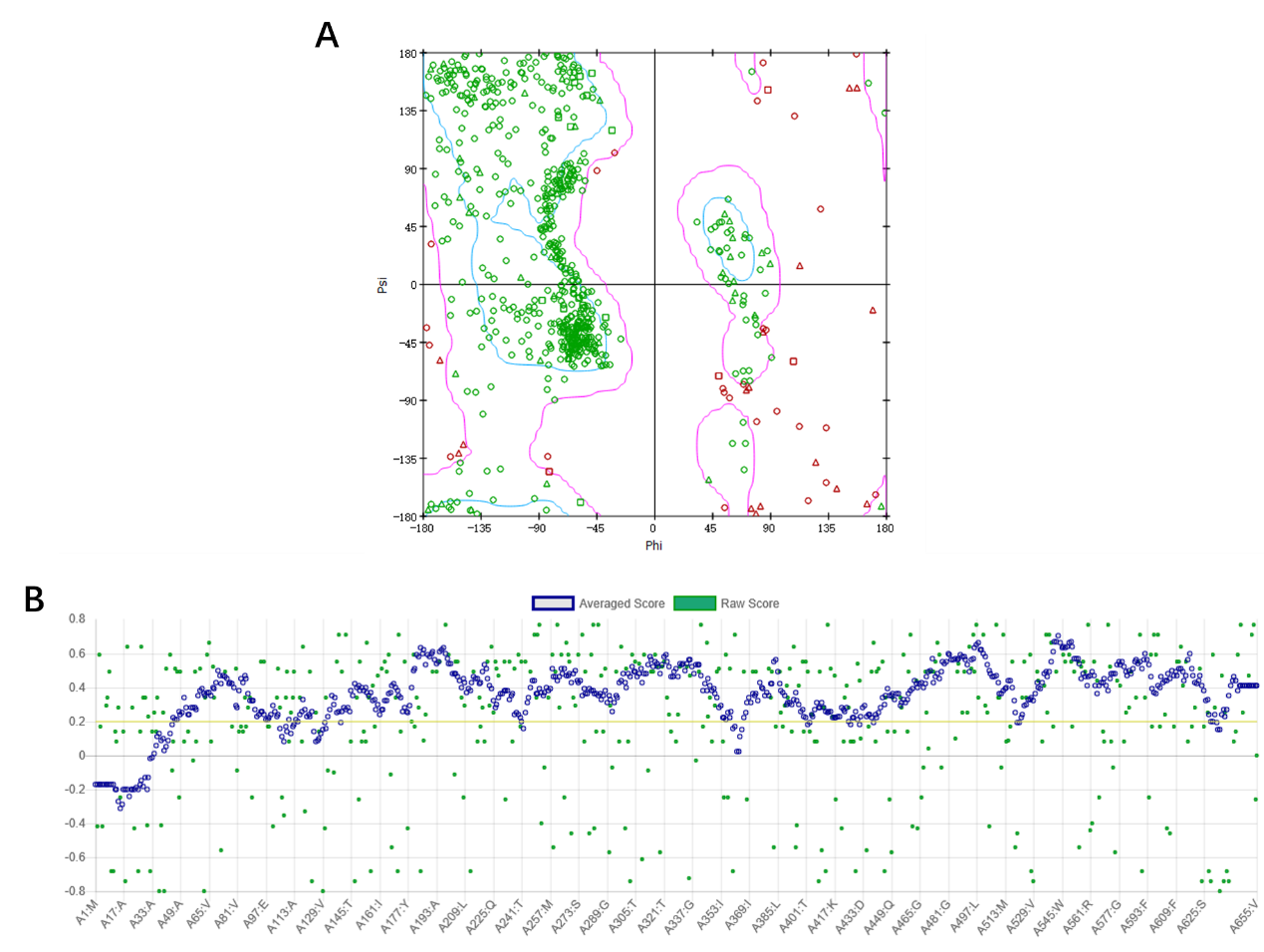


cDNA sequence of α-L-rhamnosidase N12-Rha (GenBank: KC750908.1):

atgtggtctt cctggctgct gtcggcatta ctggccactg aagcgttggc cgtaccctac

61 gaggagtaca ttctagcccc gagctctcgc gacttggctc ctgcgtccgt tcgccaggtg

121 aacggttccg tcaccaatgc ggccgctttg accggtgctg gtggacaggc cacttttaat

181 ggcgtctcgt cagtcacata cgattttggc atcaatgttg ctggtattgt gtctgtggat

241 gtcgcttccg cctcctccga gtccgccttt atcggcgtga ccttcaccga gtctagtatg

301 tggattagta acgaggcatg cgatgctacc caggatgcgg gtcttgacac tcccctctgg

361 tttgctgtcg gacagggagc gggtgtgtat tcagtgggga agaagtacac ccggggtgcc

421 ttccggtata tgacggtcgt tagcaacaca accgccacag tctccctcaa cagcgtcaag

481 atcaactata cggcatctcc catacaggac ctccgtgcat acacggggta cttccacagc

541 agtgatgaac tcctcaaccg catctggtat gccggtgcgt ataccttaca actatgcagt

601 atcgatccca ccacgggaga cgctttggtg ggactgggcg ccatcacctc gtctgagacc

661 atcacgctgc cgcagacgga caagtggtgg accaactaca ccatcaccaa tggcagcagt

721 acgttgacgg atggagccaa acgtgaccga cttgtctggc caggtgacat gtccattgct

781 ttggagagtg tagctgtcag taccgaggat ctgtatagtg tccgcacagc gttggaatct

841 ttgtatgctc ttcagaaagc cgatggccaa cttccctatg ctggaaagcc attctacgac

901 acggtcagct tcacctacca tctgcacagc ctggttggcg cggcatctta ttaccaatac

961 actggggacc gcgcgtggtt gacccggtat tggggtcagt acaagaaggg tgttcaatgg

1021 gcgttgtcgg gcgtggacag cacaggtctg gccaatatca cagccagtgc tgactggctg

1081 aggtttggca tgggggcaca taatatcgaa gcgaacgcaa tcctgtacta tgttctcaat

1141 gatgccatct ctctcgccca gtctctgaat gacaacgcac ccatcaggaa ttggactgct

1201 actgcagccc ggatcaagac agtggcaaac gaactccttt gggacgacaa gaacggactc

1261 tataccgaca acgagaccac caccctgcac ccgcaagacg gcaactcctg ggctgtcaag

1321 gcaaacctga ccctctcggc caaccagagt gccatcatct ctgaatcgct cgctgcccgc

1381 tggggcccat acggagctcc cgccccagag gcaggcgcaa cggtgtcgcc tttcatcggc

1441 ggtttcgagc tgcaggccca ctaccaggcc ggccagcccg accgcgcact tgatttactg

1501 cggttgcagt ggggattcat gctggacgac ccgcggatga ccaactcgac tttcatcgag

1561 gggtactcca cggacggatc gctggtatac gcgccgtaca ccaataggcc gcgagtgtcg

1621 cacgcgcacg ggtggtccac gggcccgacg tcagcattga ccatctacac ggccgggttg

1681 cgtgtcaccg gaccagcggg tgcgacctgg ctgtacaagc cacagccggg aaatttgacc

1741 caagttgaag ctgggtttag tacccggctg gggtcgtttg cgtcaagctt cagcagatca

1801 gggggtagat atcaggaact gtcgttcacc actccgaacg ggacgactgg ctcggtggag

1861 ctgggggatg tgagtggaca attagtctcg gaggggggag tgaaggtgca gttagtggga

1921 ggtaaggcga gtggactgca gggtgggaaa tggcggttga atgtgtaa
